# Euglycemic ketoacidosis in a non-diabetic patient with Duchenne muscular dystrophy on dapagliflozin

**DOI:** 10.1007/s11739-025-04058-3

**Published:** 2025-09-01

**Authors:** Ana María Gutiérrez-Baena, Mariona Tegido Mateu, Paula Álvarez Schlegel, Sandra Clotet-Vidal

**Affiliations:** 1 Internal Medicine Department, Hospital de La Santa Creu i Sant Pau, 08041 Barcelona, Spain; 2 Institut Català de La Salut, CAP Besòs, 08019 Barcelona, Spain

**Keywords:** Euglycemic ketoacidosis, Non-diabetic, Dapaglifozin

## Abstract

Euglycemic ketoacidosis (EKA) is an uncommon but potentially life-threatening condition characterized by ketoacidosis with normal or mildly elevated blood glucose levels. We report the case of a 24-year-old male with advanced Duchenne muscular dystrophy and cardiomyopathy treated with dapagliflozin, who developed EKA triggered by reduced food intake due to dysphagia and delayed intestinal motility. The patient presented with metabolic acidosis and elevated ketones but maintained normal glucose levels. Discontinuation of dapagliflozin, along with intravenous bicarbonate and dextrose administration, led to clinical and biochemical resolution without the need for insulin therapy. This case highlights the importance of recognizing SGLT2 inhibitor-associated EKA in non-diabetic patients, especially during fasting or stress conditions, to prevent misdiagnosis and ensure prompt management. Increased clinical vigilance and patient education are essential given the expanding use of these agents in non-diabetic populations.

## Brief clinical vignette

Euglycemic ketoacidosis (EKA) is an uncommon life-threatening emergency characterized by ketoacidosis (pH < 7.3 or serum bicarbonate < 18 mmol/L) with either normal plasma glucose or mild degree of hyperglycemia (< 14 mmol/L). EKA is a rare complication of type 1 diabetes and has become more frequent in type 2 diabetes since the introduction of sodium glucose cotransporter 2 inhibitors (SGLT2i). The absence of hyperglycemia may delay diagnosis and treatment leading to worse outcomes. The treatment is based on insulin, fluid resuscitation, and early dextrose containing fluids to avoid hypoglycemia. Although EKA is a well-known complication in diabetic patients on SGLT2i, to date. there is limited data on SGLT2i-related EKA in non-diabetic patients. We discuss the case of a non-diabetic male with advanced Duchenne muscular dystrophy and heart failure managed with dapagliflozin who presented EKA related to reduced food intake prompted by dysphagia and slowed intestinal motility. This case exemplifies how physicians must be vigilant about the risk of non-diabetic SGLT2i-related EKA during fasting and stress, especially in sarcopenic patients, to avoid misdiagnosis and promptly manage this scenario.

## Case report

We report the case of a 24-year-old male diagnosed with advanced-stage Duchenne muscular dystrophy (DMD) with dilated cardiomyopathy which led to heart failure with reduced left ventricular ejection fraction (38.4% in the biplanar measurement). His regular medication regimen included dapagliflozin 10 mg every 24 h (a SGLT2i), which was started in January 2024, enalapril 2.5 mg every 8 h, and bisoprolol 1.25 mg every 12 h.

In July 2024, the patient visited the emergency department (ED) due to an episode of abdominal pain, nausea, vomiting and decreased food intake during a respiratory infection accompanied with constipation. His vital signs were as follows: blood pressure 104/61 mmHg, heart rate 127 bpm, and oxygen saturation 94%. A physical examination revealed mild pain in the abdominal exploration. Initial tests revealed: metabolic acidosis with pH of 7.21, pCO2 27 mmHg, HCO3 10 mmol/l, potassium 3.9 mmol/l, lactate 0.8 mmol/L, and glucose 4.50 mmol/L. Further serum analysis showed no alteration of ions, renal, or hepatic function. There were signs of infection with elevated leukocytes at 13.3 × 10^9^/L with 85.3% of neutrophils and CRP at 30.6 mg/L. Suspecting aspiration pneumonia, ceftriaxone, metronidazole, and laxatives were given. Bicarbonate was administered intravenously. The patient improved with resumed intake and was discharged within 36 h.

In October 2024, the patient returned to the ED presenting abdominal discomfort due to sensation of fullness, nausea, vomiting, and persistent constipation which led to decreased food intake. Upon arrival, his vital signs were as follows: blood pressure 115/90 mmHg, heart rate 90 bpm, and oxygen saturation of 99%. A physical examination revealed a distended abdomen. Initial venous blood analysis showed a pH of 7.15, pCO2 28 mmHg, HCO3 12 mmol/L, potassium 3.7 mmol/L, and lactate 0.5 mmol/L. Blood glucose levels remained below 4.44 mmol/L, with elevated ketones (> 8.0 mmol/L). Further serum analysis showed sodium 135 mmol/L, creatinine 23 μmol/L, hemoglobin 154 g/L, platelets 520 × 10E9/L (Table 1). Liver function tests were normal, and abdominal ultrasound revealed no signs of acute pathology. The patient had no history of diabetes; therefore, a further evaluation was initiated to assess a possible diabetes onset. The results later revealed a hemoglobin A1c level of 4.8% and a C-peptide concentration of 723 pmol/L, with negative anti-IA-2 and anti-GAD-65 antibodies.

**Table 1:** Laboratory findings at the October 2024 episode

Test	Initial value	Value at discharge	Normal value
pH	7.15	7.44	7.35–7.45
Bicarbonate (mmol/L)	12.1	29.2	22–25
Anion Gap	17.6	9	8–12
Lactate (mmol/L)	0.5		0.6–2.5
Ketonemia (mmol/L)	High (> 8)	< 0.3	< 0.5
Sodium (mmol/L)	135	139	136–145
Potassium (mmol/L)	3.85	4.38	3.50–5.10
Urea (mmol/L)	1.4	3.7	3.2–7.4
Creatinine (mmol/L)	23	17	64–110
Glucose (mmol/L)	3.8	5.6	3.0–6.0
Albumin (g/L)	50.3	39.3	35–52
Alkaline phosphatase (U/L)	89		37–106
Gamma-glutamyl transferase (U/L)	19		11–59
C-reactive protein (mg/L)	19.4		0–5
Red blood cell (10^12^/L)	5.49	4.45	4.50–5.70
Hemoglobin (g/L)	154	125	130–170
White blood cell (10^9^/L)	10.68	9.24	3.80–11.00
Neutrophil (%)	82.8	48	40–70
Lymphocyte (%)	9.8	36	20–40
Platelets (10^9^/L)	520	244	140–350
Glycated hemoglobin	4.8%		4.6–5.8
C-peptide (pmol/L)	723		370–1470
Anti-GAD-65 (U/mL)	< 0.50		< 5.0
Anti-IA-2 (U/mL)	< 0.75		0–7.50

Laboratory findings at the hospital admission and during hospitalization

Given the suspicion that dapagliflozin could be a triggering factor, the medication was discontinued. Laxatives and enemas were administered, resulting in bowel evacuation and relief of fullness sensation. A total of 200 mEq/mL of bicarbonate was administered intravenously as a bolus. Despite correction of the acidosis (pH 7.39 after 16 h), ketonemia remained elevated at 5.0 mmol/L. A 10% dextrose infusion (150 g over 24 h) was administered during the emergency management, leading to normalization of analytical parameters and clinical improvement. During hospitalization, moderate to severe oropharyngeal dysphagia with pharyngeal phase impairment was noted. The patient was kept under observation for an additional 48 h with an oral diet adapted to his needs, maintaining normal pH, glucose, and ketone levels.

EKA secondary to SGLT2i is a widely recognized adverse effect in diabetic patients due to their mechanism of action [1]. By blocking glucose reabsorption in the renal proximal tubule, SGLT2i reduce plasma glucose levels and stimulate glucagon secretion from pancreatic alpha cells, promoting hepatic ketogenesis. In addition to their hypoglycemic effect, they mimic a fasting and hypoxia state, activating metabolic pathways that support cellular homeostasis, ultimately exerting nephroprotective and cardioprotective effects [1]. According to European guidelines, SGLT2i represent a fundamental pillar in the treatment of heart failure and chronic kidney disease, not requiring a diagnosis of diabetes [1].

EKA related to SGLT2i manifests similarly to hyperglycemic diabetic ketoacidosis, presenting with malaise, anorexia, tachypnea nausea, vomiting, and weight loss. The absence of pronounced hyperglycemia leads to a minor dehydration in EKA in these patients and, due to the half-life of the drug, has a longer duration. EKA related to SGLT2i presents a diagnostic challenge. Recognizing signs and symptoms of this medical emergency is essential to treat it properly. Nonetheless, the clinical approach has significant modifications compared to diabetic ketoacidosis because the pathophysiology differs. Although it has not yet been standardized, the administration of glucose and drug withdrawal are the fundamental pillars, as it was performed in this patient [2].

After an episode of EKA, SGLT2i should be temporarily discontinued, and their reintroduction after its resolution should be a personalized and shared decision. Currently, in diabetic patients, it is recommended to pause SGLT2i in acute settings that increases energy consumption as well as in scheduled fasting, for example before surgery. The management of SGLT2i in non-diabetic patients has not yet been systematized. Educating patients about the mechanisms and symptoms of EKA is key to prevent this adverse effect [3].

EKA related to SGLT2i in non-diabetic patients has also been recently reported associated to coexisting risk factors [4]. The incidence of non-diabetic SGLT2i EKA is uncommon; however, the widespread use of these agents suggests that this complication will be increasingly encountered.

Patients with DMD can experience episodes of EKA with elevated ketonemia, particularly during periods of decreased food intake. Gastric fullness, appetite loss, and constipation can lead to decreased food intake as a result from functional deterioration of the gastrointestinal smooth muscle in these patients. DMD is associated with reduced glycogen reserves due to diminished muscle mass, which impairs glycogenolysis and promotes ketogenesis [5].

In this patient, diminished glycogen reserves associated with DMD, reduced food intake, and SGLT2i induced glucosuria favored the development of EKA. The condition resolved with rapid intravenous glucose and bicarbonate administration without the need for insulin. Multidisciplinary evaluation of dysphagia was necessary to ensure safe oral intake. Avoiding prolonged fasting was recommended, and dapagliflozin was discontinued to prevent further episodes.

EKA is a rare but potential adverse effect of SGLT2i that can occur even in non-diabetic patients in situations of reduced glycogen reserves and impaired food intake. It poses a diagnostic and management challenge for clinicians and should be suspected in patients with risk factors presenting with suggestive clinical features. Given the increased use of SGLT2i, it is essential for clinicians to recognize this potential adverse effect, even in individuals without diabetes. Protocols recommend discontinuing SGLT2i in high-risk ketogenic situations. Physicians must remain vigilant and educate patients on the risks and warning signs of EKA to reduce misdiagnoses and improve patient outcomes.

## Data Availability

The data underlying this case report are available in the published article.
